# Procedural Competency in Academic Emergency Medicine Attending Physicians: How Is Competency Maintained and Evaluated by Academic Institutions in the US?

**DOI:** 10.7759/cureus.16719

**Published:** 2021-07-29

**Authors:** Erin Bell, Michelle A Fischer, Haley Sinatro

**Affiliations:** 1 Emergency Medicine, Penn State College of Medicine, Hershey, USA

**Keywords:** procedural skills, competency, simulation, skills maintenance, skills decay

## Abstract

Introduction

There have been numerous studies examining the minimum graduation requirements for resident training of procedural skills within Emergency Medicine programs; however, how academic medical centers in the United States maintain Emergency Medicine attending procedural skill competency has not been explored.

Objectives

The aim of this study was to examine the processes in place to evaluate and track the procedural skills practices of Emergency Medicine attending physicians at academic institutions in the US.

Methods

An exploratory cross-sectional survey was sent to all 39 ACGME-accredited Emergency Medicine programs in the US through a REDCap survey in 2020. Survey items inquired about the current methods in place to maintain competence on 13 procedural skills performed by Emergency Medicine providers.

Results

The survey response rate was 26.9%. The majority of programs did not have a process in place to evaluate procedural skills at the time of initial appointment (74.3%), and almost half of participating programs reported no formalized process during employment (51.3%). Institutions reported no minimum required number for the following procedures: dislocation reduction, intraosseous placement, lateral canthotomy, lumbar puncture, paracentesis, pericardiocentesis, thoracentesis, transvenous cardiac pacing, and tube thoracostomy. For central venous access, cricothyrotomy, endotracheal intubation, and procedural sedation, 25.6% or less of institutions had minimum annual requirements.

Conclusion

This study summarized the current methods in place to assess Emergency Medicine attending procedural skills at US academic institutions and demonstrated that the majority of programs lack a formalized method to assess attending procedural competency. Further research is needed to determine the value and benefit of different methods available for procedural skill competency assessment. It is believed that preventing procedural skill decay in attending physicians by a standardized process has the potential to improve patient outcomes, reduce costs and complication rates, and improve physician self-esteem, well-being, and confidence.

## Introduction

Procedural skill competency is the backbone of Emergency Medicine, but what is competency? In the medical field, it is typically interpreted as an individual having not only a set of demonstrable characteristics but also those skills necessary to complete a task successfully and efficiently. Competency is the minimum threshold or standard for a practicing physician. The commonly accepted pathway for procedural competency is: Learn, See, Practice, Prove, Do, and Maintain [1-2]. Proficiency, on the other hand, is mastery [3]. Emergency Medicine attending physicians at academic training programs are expected to possess some degree of mastery of the clinical practice of emergency medicine. Emergency Medicine is a specialty that not only requires cognitive aptitude but also procedural expertise. Maintaining procedural skills can be challenging, particularly in an academic setting where it is often a resident learner, rather than the attending, performing the procedures under the watchful guidance of the mentor.

As per current American College of Graduate Medical Education (ACGME) guidelines, Emergency Medicine Residency programs in the United States must meet minimum requirements for resident training based upon the EM Model for clinical care which includes medical knowledge, patient care, and procedural skills [4]. These resident requirements and strategies, within and outside of the United States, have been well studied and documented [5].

Most Emergency Medicine Residency programs have determined a list of procedures along with the minimum quantity performed needed for residency graduation. The American College of Emergency Physicians (ACEP) released guidelines regarding how certain clinical privileges and procedures are recommended to be credentialed and delineated with physicians of different levels of training [6]. It was the belief that establishing criteria for proficiency and establishing numerical thresholds for procedures may be difficult to track due to the range and infrequency of certain procedures [6]. Further, it is not clear whether such tracking of urgent/emergent procedural skills is a valid component of proficiency assessment [2]. How these guidelines have been applied in specific medical centers or how these centers ensure and record the procedural skill competency of their attending physicians has not been explored in previous literature.

This study seeks to evaluate the current measures in place that evaluate and track the procedural skills practices of emergency medicine attending physicians working at academic institutions with emergency medicine residency programs across the US. This is an exploratory study to gather both qualitative and quantitative data on current processes, as well as percentages of completed procedures. We hypothesize that a standardized process across US academic medical centers to evaluate emergency medicine attending physician procedural skills does not currently exist. The maintenance of procedural skills of academic emergency medicine attending physicians has never been explicitly studied before despite these clinical skills being a significant component of clinical practice. By having a better understanding of how academic medical institutions across the United States monitor attending physician procedural skills, one can then adopt an institution-specific and individualized process for attending procedural skills training and professional development. The belief is that this examination of internal procedural review has the potential to have a positive downstream effect of not only increased physician proficiency and competence but also improved patient safety reduced medical error and perhaps even improved emergency department efficiency. 

## Materials and methods

There are currently over 200 ACGME-accredited Emergency Medicine Residency programs in the United States. Programs were identified via the Fellowship and Residency Electronic Interactive Database (FREIDA). The contact information for 145 Emergency Medicine Chairs and Emergency Medicine Residency program coordinators was obtained from the department’s website and contacted via telephone and/or email. A REDCap survey was created and distributed via email to the points of contact. If no response was received, a follow-up email was sent. If there was no response from the follow-up email, the potential participant received a phone call and a subsequent survey resend. This study was approved by the Institutional Review Board (IRB).

## Results

Of the 145 programs surveyed, 39 responses were received, with a response rate of 26.9%. 

Existing institutional processes

The first series of survey questions examined the current institutional processes in place to evaluate procedural skills upon hire if a different process was in place for new graduate resident hires, the process used for procedural skills maintenance, and if a minimum number of procedures was necessary for hire. Of the 39 institutions that responded to the survey, the majority of programs (74.3%) did not have a process in place to evaluate procedural skills at the time of hire (Table 1). Only two institutions, or 5% of the institutions surveyed, reported that they had different considerations for evaluating procedural skills at hire for attending physicians of different experience levels, however, it is not specified whether these considerations are part of a formal process. The majority of programs (51.3%) did not have a formalized process for procedural skill evaluation. Over 20% of the institutions stated that processes other than simulation, procedure logs, or annual training were used for procedural skills evaluation. Some of these methods included using billing codes, optional simulations, reviewing videos of resuscitations, requiring a minimum number of clinical shifts, performance evaluations, and ongoing training. A few institutions had procedure logs and formal annual training as the method for their procedural skills evaluation.

**Table 1:** Institutional Procedural Competency Evaluation Methods

	Total (N=39)
Processes to Evaluate Procedural Skills When Hiring	
Recommendations	29 (74.3%)
None	6 (15.4%)
Simulations	2 (5.1%)
Internal hiring	1 (2.6%)
Procedure lists	1 (2.6%)
Different Considerations to Evaluate Skills When Hiring New Graduates vs. Experienced Hires	
No	37 (94.9%%)
Yes	2 (5.1%)
Processes to Ensure Continued Skills Competency Of Attending Physicians	
No formalized process	20 (51.3%)
Other	8 (20.5%)
Simulation	7 (17.9%)
Procedure logs	3 (7.7%)
Annual training	1 (2.6%)
Minimum Number of Independently Completed Procedures	
No	28 (71.8%)
Yes	11 (28.2%)

Minimum annual number of attending procedural requirements

The second part of the survey investigated if institutions had a minimum annual quota for common and/or critical attending procedural skills (Table 2). There was no reported minimum required number for the following procedures: dislocation reduction, intraosseous placement, lateral canthotomy, lumbar puncture, paracentesis, pericardiocentesis, thoracentesis, transvenous cardiac pacing, and tube thoracostomy. A minority of institutions, only 7%, had annual minimum requirements for central venous access procedures, with a mean of 6, and a range of 2-10. One institution had a minimum number of one annual cricothyrotomy performed via simulation. Ten percent (10%) of institutions had minimum annual requirements for intubations, with a mean of 5.8, and a range of 2-10. Over a quarter of institutions surveyed (25.6%) had a minimum annual requirement for procedural sedations with a mean of 7 and a range of 2-20.

**Table 2:** Minimum Annual Attending Procedure Requirements

Procedure	N=39	Mean (range)
Central Venous Access	3 (7.7%)	7.3 (2-10)
Cricothyrotomy	1 (2.6%)	1 (0-1)
Intubations	4 (10%)	5.8 (2-10)
Procedural Sedation	10 (25.6%)	7 (2-20)
Dislocation Reduction	0 (0.0%)	NA
Lateral Canthotomy	0 (0.0%)	NA
Lumbar Puncture	0 (0.0%)	NA
Paracentesis	0 (0.0%)	NA
Pericardiocentesis	0 (0.0%)	NA
Intraosseous Placement	0 (0.0%)	NA
Thoracentesis	0 (0.0%)	NA
Transvenous Cardiac Pacing	0 (0.0%)	NA

## Discussion

The maintenance of procedural skills of academic emergency medicine attending physicians has never been explicitly studied before despite these clinical skills being a significant component of clinical practice. By having a better understanding of how academic medical institutions across the United States monitor attending physician procedural skills, one can then adopt an institution-specific and individualized process for attending procedural skills training and professional development. The belief is that this examination of internal procedural review has the potential to have a positive downstream effect of not only increased physician proficiency and competence but also improved patient safety, reduced medical error, and perhaps even improved emergency department efficiency.

Our results indicate that most institutions in academic Emergency Medicine do not have a standardized process in place for maintaining procedural skills in attending physicians. Of the 39 institutions that responded to the survey, the majority of programs (74.3%) did not have a process to evaluate procedural skills at the time of hire. Responding institutions recorded procedural skills processes utilized included simulations (17.9%), procedure logs (7.7%), and annual training (2.6%). Institutions had no reported minimum required for a dislocation reduction, intraosseous placement, lateral canthotomy, lumbar puncture, paracentesis, pericardiocentesis, thoracentesis, transvenous cardiac pacing, and tube thoracostomy. Several programs reported internal committees at their institutions that assess attending procedural skill competency annually through simulation centers or cadaver labs. Others programs reported that an attending’s procedural skills competency was assessed only if concerns were reported about that individual. Interestingly, the majority of institutions expressed the desire to implement a mechanism to assess attending procedural competency.

Several studies examining the role of a cadaver, simulation center, or animal laboratory training in resident procedural skills training found these techniques to be quite effective. A simulation-based mastery learning program increased resident procedural skills in simulated central venous catheter insertion and decreased complications related to the procedure in actual patient care [7]. Animal laboratory training for residents increases procedural competency and speed in performing resuscitative procedures [8]. Such studies leave little doubt of the utility of animal labs, simulation centers, or other similar measures that improve procedural skills in resident learners. A pediatric emergency medicine survey that looked into existing processes for maintaining procedural competency found that while competency is perceived as important, there was a discrepancy regarding what training what necessary to maintain clinical skills [9]. Preserving procedural competency is essential, as it will prevent skill decay. Skill decay is defined as the loss of acquired skills after non-use [10]. By retaining procedural skill competency, skill decay can be avoided or at least minimized. This becomes an essential concept, as preservation of procedural skills will increase provider confidence, has positive effects on patient safety, and has the potential to result in an overall increase in the quality of patient care. Very few studies have looked at the level of training, and/or frequency or quantity of performed procedures by the operator with procedural complications completed in the emergency room. One such study, conducted in Canada, surveyed Emergency Medicine physicians about procedural skills competency and found that confidence levels increased when more procedures were performed [11]. Further research is indicated to determine the best structure to utilize to maintain the procedural skills of veteran emergency medicine physicians, and how this system would be structured.

Limitations

Although there are over 200 Emergency Medicine Residency programs, only 145 programs were able to be surveyed, and despite multiple attempts, only 39 responses were received with an overall response rate of 26.9%. This low response rate does lend itself to sampling bias. We hypothesize that the lower response rate may be due to the nature of the research, which involved asking programs to reflect on internal standards for procedural competency, often an area of contention among the specialty. Contacting the programs was challenging, and information was often relayed through administrative assistants or program coordinators, not the department chairs themselves, lending itself to receiving less accurate or factual data. Future investigation is needed to determine if causality truly exists between skills decay and procedural complication rates.

## Conclusions

Our aim with this study was to elucidate whether current existing standards for Emergency Medicine attending physician procedural competency exist at US academic medical centers. The results of this study demonstrate that there is no current standard or guideline in place at those programs that responded. With the knowledge of skill decay, Emergency Medicine attending physicians must be proactive and creative. We are naturally active lifelong learners and this same principle must be applied to our procedural skills. Practice, with real-time guidance and feedback, can minimize and even eliminate skill decay, particularly for those procedures performed infrequently such as cardiac pacing or lateral canthotomy. By assuming the role of an active learner, we have the potential to not only improve our patient outcomes and complication rates but also enhance our self-confidence. Further research is needed to determine to what degree skill decay influences complication rates, and how a system assessing procedural competency may influence these rates. While the focus of this study is Emergency Medicine, procedural competency applies to all fields of medicine.
